# HIV prevention among marginalized adolescents: PrEP1519 results and perspectives

**DOI:** 10.11606/s1518-8787.2024058supl1ed

**Published:** 2024-10-11

**Authors:** João Luiz Bastos, Tonantzin Ribeiro Gonçalves, Eliseu Alves Waldman

**Affiliations:** I Simon Fraser University. Faculty of Health Sciences. Burnaby, BC, Canada Simon Fraser University Faculty of Health Sciences BC Burnaby Canada; II Universidade Federal de Ciências da Saúde de Porto Alegre. Departamento de Psicologia. Porto Alegre, RS, Brasil Universidade Federal de Ciências da Saúde de Porto Alegre Departamento de Psicologia RS Porto Alegre Brasil; III Universidade de São Paulo. Faculdade de Saúde Pública. Departamento de Epidemiologia. São Paulo, SP, Brasil Universidade de São Paulo Faculdade de Saúde Pública Departamento de Epidemiologia SP São Paulo Brasil

As one of the main strategies currently available to prevent HIV, pre-exposure prophylaxis (PrEP) has been adopted in Brazil since 2017 in the context of a broader approach for populations at high risk of infection. The high efficacy and relative ease of implementation have motivated the use of PrEP in varied contexts around the world. However, the absence of Brazilian guidelines to implement PrEP among adolescents under 18 years of age supported the PrEP1519 study from 2018 onwards. This cohort was conducted in three state capitals in Brazil (Belo Horizonte, São Paulo, and Salvador) with male adolescents who have sex with men (aMSM) and adolescent transsexual women and *travestis* (aTWT) aged 15-19 years to determine the effectiveness of PrEP among these populations^1^.

Since its conception, PrEP1519 was designed following an interdisciplinary and multiprofessional perspective based on the collection of both qualitative and quantitative data. The study coordinators also prioritized the recruitment of a diverse sample of participants, considering gender identity, class, race, and experiences of violence or discrimination. This commitment to diversity and inclusion ensured that the study results represented and were relevant to the populations most vulnerable to HIV infection.

The engagement between researchers, community members, and healthcare providers, among others, was key to the knowledge production and publication of academic papers with great relevance to address the HIV/AIDS epidemic in Brazil. Analyses on the acceptability of PrEP^2^, the strategies to recruit adolescents at high risk of infection^3^, the frequency of a range of sexually transmitted infections (including HIV)^4^, the use of HIV preventive methods^5^, and the construction of links between health services and target populations^6^ have been published in various media, including specialized scientific journals. These publications not only expand scientific knowledge, but also provide practical guidelines for the effective implementation of HIV prevention programs.

This supplement of *Revista de Saúde Pública* builds on an additional and no less important set of PrEP1519 findings. Consistent with its broad interdisciplinary and methodological origins, the reader will find a collection of quantitative and qualitative studies on a variety of topics. While quantitative studies focus on the prevalence of hepatitis and predictors of condomless anal sex in aMSM and aTWT, qualitative analyses address the use of an intersectional perspective to investigate HIV prevention, social representations of HIV and care in newly diagnosed youths, and the categories “risk” and “pleasure” in affective-sexual relationships protected by PrEP use.

This supplement also includes studies on the perceptions and practices of healthcare providers who follow the administration of PrEP and the challenges faced by adolescents who use PrEP on demand. These studies are crucial to understanding the barriers and facilitators in PrEP adoption, enabling interventions to be tailored to better meet the adolescents’ needs. Given that the PrEP1519 spanned the COVID-19 pandemic, its impact on the relationships aTWT establish with PrEP services was also considered. Despite its additional challenges, the pandemic offered opportunities to innovate and adapt health services to continuously provide essential care.

Together, the studies in this supplement provide original and essential knowledge to address the HIV/AIDS epidemic in adolescents under 18, especially aMSM and aTWT. The focus on multiply marginalized populations is undoubtedly a strength of the study, promoting the visibility of populations that are often made invisible, including their specific health care needs and strategies to address them. Initiatives such as PrEP1519 enable the improvement of health policies by combating rather than exacerbating social injustices in the frequency and distribution of health issues.

The editorial board of *Revista de Saúde Pública* is very proud and grateful for the opportunity to support the dissemination of these works, thus contributing to the promotion of health and the mitigation of health inequities. We hope this supplement will inspire new studies and public policies that continue to further HIV prevention and care for the most vulnerable populations.
