# Supplementary material for: HIV prevention among marginalized adolescents: PrEP1519 results and perspectives
Source: Rev Saude Publica. 2024 Oct 11;58(Suppl 1):1s. doi: 10.11606/s1518-8787.2024058supl1ed (PMC11573369; doi:10.11606/s1518-8787.2024058supl1ed)
Supplement: Translation

# Translation: Prevenção do HIV entre adolescentes marginalizados: resultados e perspectivas do PrEP1519

**Authors:** João Luiz Bastos, Tonantzin Ribeiro Gonçalves, Eliseu Alves Waldman

**Affiliations:** I Simon Fraser University. Faculty of Health Sciences. Burnaby, BC, Canada Simon Fraser University Faculty of Health Sciences BC Burnaby Canada; II Universidade Federal de Ciências da Saúde de Porto Alegre. Departamento de Psicologia. Porto Alegre, RS, Brasil Universidade Federal de Ciências da Saúde de Porto Alegre Departamento de Psicologia RS Porto Alegre Brasil; III Universidade de São Paulo. Faculdade de Saúde Pública. Departamento de Epidemiologia. São Paulo, SP, Brasil Universidade de São Paulo Faculdade de Saúde Pública Departamento de Epidemiologia SP São Paulo Brasil

Considerada uma das principais medidas atualmente disponíveis para a prevenção do HIV, a profilaxia pré-exposição (PrEP) tem sido adotada desde 2017 no Brasil como parte de uma estratégia mais ampla para populações em alto risco de infecção. A alta eficácia e a relativa facilidade de implementação têm motivado o emprego da PrEP nos mais variados contextos e situações ao redor do mundo. No entanto, a ausência de diretrizes brasileiras para a implementação da PrEP junto a adolescentes menores de 18 anos de idade embasou a realização do estudo PrEP1519 a partir de 2018. Essa pesquisa é uma coorte conduzida em três grandes cidades do país (Belo Horizonte, São Paulo e Salvador) com adolescentes homens que fazem sexo com homens (AHSH) e adolescentes mulheres transexuais e travestis (AMTrT), entre 15–19 anos de idade, que visou determinar a efetividade da PrEP nesses segmentos populacionais^1^.

Desde o início, em 2018, o PrEP1519 foi concebido a partir de uma perspectiva interdisciplinar e multiprofissional, fundamentado na produção e análise de dados qualitativos e quantitativos. Também foi uma prioridade para os idealizadores da investigação o recrutamento de uma amostra diversa de participantes, considerando identidade de gênero, classe, raça e experiências de violência ou discriminação. Esse compromisso com a diversidade e a inclusão assegurou que os resultados obtidos fossem representativos e relevantes para as populações mais vulneráveis à infecção pelo HIV.

O engajamento entre pesquisadores, membros da comunidade, profissionais de saúde, entre outros, tem permitido a produção de conhecimento e a divulgação de publicações de alta relevância acadêmica e política para o enfrentamento da epidemia de HIV/Aids no Brasil. Análises sobre a aceitabilidade da PrEP^2^, estratégias para alcançar e recrutar adolescentes em alto risco de infecção^3^, a frequência de uma gama de infecções sexualmente transmissíveis (incluindo o HIV)^4^, o uso de métodos preventivos do HIV^5^ e a construção de vínculos entre serviços de saúde e as populações-alvo do estudo^6^ já foram veiculadas em diversos meios de comunicação, incluindo periódicos científicos especializados. Essas publicações não apenas expandem o conhecimento científico, mas também fornecem diretrizes práticas para a implementação eficaz de programas de prevenção do HIV.

Neste suplemento da Revista de Saúde Pública, enfatiza-se um conjunto adicional e não menos importante de achados do PrEP1519. Coerente com as origens interdisciplinares e metodológicas amplas da pesquisa, o leitor encontrará nas páginas seguintes uma coleção de trabalhos quantitativos e qualitativos que abrangem uma variedade de questões de interesse. Enquanto os estudos quantitativos se debruçam sobre, por exemplo, a prevalência de hepatites e os preditores do sexo anal sem preservativo em AHSH e AMTrT, as análises qualitativas abordam o emprego de uma perspectiva interseccional na investigação da prevenção do HIV, as representações sociais do HIV e do cuidado em jovens recentemente diagnosticados, bem como as categorias “risco” e “prazer” no contexto de relações afetivo-sexuais protegidas pelo uso do PrEP.

Compõem igualmente o suplemento, estudos sobre as percepções e práticas dos profissionais de saúde que acompanham a administração da PrEP e os desafios enfrentados pelos adolescentes que fazem uso da PrEP sob demanda. Esses estudos são cruciais para entender as barreiras e facilitadores na adoção da PrEP, permitindo que intervenções sejam adaptadas para melhor atender às necessidades dos adolescentes. Tendo em vista que o PrEP1519 atravessou o período da pandemia de covid-19, o impacto desta nas relações que AMTrT estabelecem com os serviços de PrEP também foi objeto de consideração. A pandemia trouxe desafios adicionais, mas também oportunidades para inovar e adaptar os serviços de saúde para continuar a fornecer cuidados essenciais.

Tomados em conjunto, os artigos incluídos neste suplemento aportam conhecimentos originais e imprescindíveis ao enfrentamento da epidemia de HIV/Aids entre adolescentes menores de 18 anos, particularmente AHSH e AMTrT. O enfoque nas populações multiplamente marginalizadas é, sem dúvida, um destaque da pesquisa, permitindo dar visibilidade a populações que muitas vezes são invisibilizadas, incluindo suas necessidades específicas e as estratégias efetivas para abordá-las. São iniciativas como o PrEP1519 que permitem aprimorar nossas políticas de saúde e melhor orientá-las para que sejam mais inclusivas e benéficas para todo o conjunto da população, combatendo e não exacerbando as injustiças sociais na frequência e distribuição de agravos e condições de saúde.

A editoria da Revista de Saúde Pública sente-se muito orgulhosa e grata pela oportunidade de colaborar com a divulgação dos referidos trabalhos, contribuindo assim para a promoção da saúde e a mitigação das injustiças sociais em saúde coletiva. Esperamos que este suplemento inspire novos estudos e políticas públicas que continuem a avançar na prevenção do HIV e no cuidado com as populações mais vulneráveis.
